# Stromal cell markers are differentially expressed in the synovial tissue of patients with early arthritis

**DOI:** 10.1371/journal.pone.0182751

**Published:** 2017-08-09

**Authors:** Ivy Y. Choi, Olga N. Karpus, Jason D. Turner, Debbie Hardie, Jennifer L. Marshall, Maria J. H. de Hair, Karen I. Maijer, Paul P. Tak, Karim Raza, Jörg Hamann, Christopher D. Buckley, Danielle M. Gerlag, Andrew Filer

**Affiliations:** 1 Division of Clinical Immunology and Rheumatology, Academic Medical Center, University of Amsterdam, Amsterdam, the Netherlands; 2 Department of Experimental Immunology, Academic Medical Center, University of Amsterdam, Amsterdam, the Netherlands; 3 Rheumatology Research Group, Institute of Inflammation and Ageing, The University of Birmingham, United Kingdom; 4 Sandwell and West Birmingham Hospitals NHS Trust, Birmingham, United Kingdom; 5 University Hospitals Birmingham NHS Foundation Trust, Birmingham, United Kingdom; Universite de Nantes, FRANCE

## Abstract

**Introduction:**

Previous studies have shown increased expression of stromal markers in synovial tissue (ST) of patients with established rheumatoid arthritis (RA). Here, ST expression of stromal markers in early arthritis in relationship to diagnosis and prognostic outcome was studied.

**Methods:**

ST from 56 patients included in two different early arthritis cohorts and 7 non-inflammatory controls was analysed using immunofluorescence to detect stromal markers CD55, CD248, fibroblast activation protein (FAP) and podoplanin. Diagnostic classification (gout, psoriatic arthritis, unclassified arthritis (UA), parvovirus associated arthritis, reactive arthritis and RA), disease outcome (resolving vs persistent) and clinical variables were determined at baseline and after follow-up, and related to the expression of stromal markers.

**Results:**

We observed expression of all stromal markers in ST of early arthritis patients, independent of diagnosis or prognostic outcome. Synovial expression of FAP was significantly higher in patients developing early RA compared to other diagnostic groups and non-inflammatory controls. In RA FAP protein was expressed in both lining and sublining layers. Podoplanin expression was higher in all early inflammatory arthritis patients than controls, but did not differentiate diagnostic outcomes. Stromal marker expression was not associated with prognostic outcomes of disease persistence or resolution. There was no association with clinical or sonographic variables.

**Conclusions:**

Stromal cell markers CD55, CD248, FAP and podoplanin are expressed in ST in the earliest stage of arthritis. Baseline expression of FAP is higher in early synovitis patients who fulfil classification criteria for RA over time. These results suggest that significant fibroblast activation occurs in RA in the early window of disease.

## Introduction

Rheumatoid arthritis (RA) is a chronic inflammatory disease affecting synovial tissue (ST) in multiple joints leading to joint destruction, deformity and disability [1]. Classifying patients in an early stage of the disease is important, as early appropriate treatment can reduce or even prevent joint destruction [2]. Paradoxically, it is in the first three months of symptoms, when optimal treatment most significantly improves outcomes, that patients with early symptoms are most difficult to diagnose and many therefore remain unclassified [3–5], resulting in a delay in optimal treatment. There is therefore a need for new diagnostic and prognostic markers. Evidence is starting to emerge that synovial tissue may be a valuable source of potential biomarkers in the earliest stages of disease when diagnosis is unclear [6].

Stromal cells play an important role in organising the structure of ST by producing extracellular matrix components, recruiting infiltrating immune cells and secreting inflammatory mediators. Furthermore, considerable evidence exists supporting a role for these cells in driving the persistence of inflammation and joint damage in RA [7–9]. The addition of novel stromal markers to existing markers such as VCAM-1, CD55 and cadherin-11 has resulted in an increasing ability to delineate subpopulations of fibroblast-like cells in the synovium that may possess differing functional properties and act as both biomarkers of outcome and novel therapeutic targets [10].

Previous work has suggested that the synovial compartment of patients with short duration RA is rich in stromal growth factors [11, 12]. In patients with early RA, concentrations of basic fibroblast growth factor and epidermal growth factor in synovial fluid are significantly higher compared to both patients with early arthritis developing non-RA diseases and patients with longer duration established RA (11). We therefore hypothesised that subpopulations within the stromal compartment might become activated and expanded during the inflammatory processes occurring in early disease, defining a profile that may be specific for RA. We chose to examine an established intimal lining layer stromal marker (CD55) [13, 14], and more recently discovered markers described both in RA and cancer, where the role of tumour-associated fibroblasts has become prominent. These include CD248 [15, 16], a synovial sublining glycoprotein marker expressed in perivascular and cancer stromal cells and in the RA synovium; fibroblast activation protein (FAP) [17–19], a cell surface protein that is highly expressed in established RA synovium with ectoenzyme activity and an important role in epithelial cancers; and podoplanin (gp38) [20–22], an intimal lining layer glycoprotein marker with roles in lymph node stromal networks and epithelial to mesenchymal transition. Using the combined resources of two collaborating cohorts with aligned data and tissue sample collection protocols, we tested the hypotheses that tissue stromal markers measured during the first three months of symptom duration would (a) differentiate diagnostic groups, and (b) distinguish between prognostic outcomes of persistence and resolution. We also examined whether expression of stromal markers correlated with clinical and ultrasonographic measures of disease activity.

## Methods

### Cohorts and synovial tissue

Synovial tissues and clinical outcome data of patients included in two early arthritis cohorts in Amsterdam (Synoviomics) and Birmingham (BEACON) were used in this study [23, 24]. Overlap of procedures for clinical data collection in the cohorts allowed comparison of all key data variables. During establishment of ultrasound guided biopsy techniques in Birmingham in 2007, standard operating procedures for tissue collection and processing were harmonised with the Synoviomics project, leading to a common protocol for tissue recovery, processing and storage. All patients were naïve to treatment with disease-modifying antirheumatic drugs (DMARDs) and corticosteroids at inclusion.

Patients with clinical synovitis present in at least one synovial joint (of 66 joints examined) were recruited to the BEACON cohort if symptom duration, defined as any symptom attributed by the assessing rheumatologist to inflammatory joint disease (pain, stiffness and/or swelling) was no greater than 3 months [25, 26]. Consenting patients underwent ultrasound guided synovial biopsy of an inflamed joint at baseline. Ultrasound guidance was used to introduce a single portal through which tissue was sampled using 2.0mm cutting edged flexible forceps (knee and ankle) or a 16G core biopsy needle (metacarpophalangeal joint) [6, 24]. Diagnostic and prognostic outcomes were assigned after 18-months of follow-up, with visits at 1, 2, 3, 6, 12 and 18 months. In Birmingham patients the joint to be biopsied was scanned immediately prior to the procedure using a Siemens Acuson Antares scanner (Siemens PLC, Bracknell, UK) and multifrequency (5-13MHz) linear array transducers. Findings of synovitis and power Doppler (PD) positivity were defined using current consensus OMERACT definitions [27]. Greyscale synovial hypertrophy and Power Doppler ultrasound variables were graded on 0–3 semi-quantitative scales as previously reported [28].

In the Synoviomics cohort [23], patients presenting with arthritis of at least one knee, ankle or wrist joint with duration of less than 1 year were included [29]. Arthritis duration was defined as the time from the first clinical signs of arthritis, irrespective of which joint was initially affected, determined by an experienced rheumatologist; data on symptom duration were also recorded, enabling comparison of patients between cohorts. At inclusion, synovial tissue was collected during a mini-arthroscopy procedure as previously described [30]. Diagnostic and prognostic outcomes were assigned after 2 years of follow up with minimum study visits at 6 months, year 1 and year 2.

VERA was defined as any patient meeting RA criteria either at baseline or cumulatively during subsequent study visits. In both cohorts, patients were classified as having RA according to the 2010 ACR/EULAR classification criteria for RA [25, 26], psoriatic arthritis (PsA) according to the CASPAR criteria for PsA [31], parvovirus arthritis based on clinical diagnosis plus serological testing, and other diagnoses by characteristic clinical features, including the presence of a pre-existing infectious episode (reactive arthritis) and uric acid crystals in synovial fluid (gout). Patients were classified as having unclassified arthritis (UA) if they did not meet any classification criteria. Patients were classified as having self-limiting disease if they had no clinical evidence of synovial swelling and had not taken DMARDs or received glucocorticoid treatment in any form in the preceding 3 months.

We included ST of seven “non-inflammatory” control individuals with joint pain and normal MRI imaging who underwent exploratory arthroscopy during which no evidence of synovial pathology was found macroscopically or on subsequent histological analysis.

All studies were approved by local medical ethical committees and patients gave written, informed consent to participate.

### Tissue staining, immunofluorescence and confocal microscopy

Synovial tissue samples were snap-frozen in Tissue-Tek OCT medium (Miles, Elkhart, IN) immediately after collection. In order to account for heterogeneity, six to eight biopsies from different areas of the joint were combined in one block of tissue. Cryostat sections (5μm) were cut, mounted on Star Frost adhesive glass slides (Knittelplaser, Baunschweig, Germany) and stored at -80°C.

All tissue staining, image acquisition and quantification took place in Birmingham and was blinded to clinical outcomes. Prior to use, slides were thawed at room temperature (RT) for 30 min. After fixation in acetone the sections were washed in PBS and blocked with 10% normal human serum for 30 min at RT. Incubation with primary antibodies was performed overnight at 4°C in blocking solution. As primary antibodies, anti-CD55 (mouse IgG2a, clone BU84; University of Birmingham, UK), anti-CD248 (mouse IgG1 supernatant, clone B1 35.1), anti-FAP (mouse IgG1, clone F11-24; eBioscience), anti-podoplanin (mouse IgG1, clone D2-40; AbD Serotec, Kidlington, UK), anti-CD31 (endothelial cell marker; mouse IgG2a, clone HEC7; Thermo Scientific, Loughborough, UK), anti-CD68 (macrophage marker; mouse IgG2b, clone Y1/82A; BD Pharmingen, Oxford, UK) were used. Tissue sections were incubated for 1 hour at RT and bound primary antibodies were detected with goat antibodies against mouse IgG1 conjugated with fluorescein (FITC), IgG2a conjugated with rhodamine (TRITC) and IgG2b conjugated with cyanine 5 (Cy™ 5) (all Southern Biotech, Birmingham, AL). To increase signal from FITC-channel, slides were stained for 30 min at RT with goat anti-FITC Alexa-488 antibody (Invitrogen, Paisley, UK). All sections were co-stained with Hoechst solution (Sigma-Aldrich Company Ltd., Gillingham, UK) to visualize cell nuclei. As a negative control, a mixture of anti-IgG1, anti-IgG2a and anti-IgG2b secondary fluorochrome-conjugated antibodies followed by anti-FITC Alexa-488 were applied to the sections after omission of the primary antibodies. Images were acquired from 1–8 different regions of each tissue section, using a Zeiss LSM 510 confocal scanning microscope and ZEN pro 2011 imaging software (Zeiss, Welwyn Garden City, UK). Settings within one staining experiment remained unchanged. For each image, the number of pixels with intensity from 30 to 255 of every fluorescent channel was quantified with ZEN pro 2011 and divided by a manually defined area (μm^2^) only including tissue zones containing cells. The average number of fluorescent pixels with intensity 30–255 per unit area (pixel/UA) from all images within one synovial tissue section was calculated. In addition, two researchers independently assessed the fluorescence level of every marker using a semiquantitative scoring system of grade 0–4 combining staining intensity and number of positive cells. Semiquantitative scores correlated well with unbiased pixel analysis scores (Spearman’s rho >0.7, p <0.001 for all markers; data not shown). In order to assess differences in expression of the stromal markers FAP and podoplanin in different anatomical regions, lining and sublining regions were identified and pixel counts individually quantified in 15 randomly selected samples from each of the early RA and non-RA groups by a blinded observer.

During the study, tissues were stained for immunofluorescence on three separate occasions in total, all performed in the Birmingham unit. In order to measure and account for any variation between staining of sections during different staining runs, we stained sections from the same 11 patients on each occasion for a range of four stromal (CD55, CD248, FAP and podoplanin) and two cellular (CD90, CD68) markers. Intraclass correlation coefficients (ICC) for markers across runs were as follows reflecting good (ICC >0.7) internal consistency for all markers except CD248, which closely approached this level (ICC 0.69): CD55: ICC 0.77; FAP: ICC 0.74; podoplanin: ICC 0.74; CD31: ICC 0.89; CD68: ICC 0.96.

For immunohistochemical staining, acetone fixed slides were re-hydrated in PBS for 10 minutes prior to blocking with Bloxall reagent (Vector Laboratories, California USA) for ten minutes followed by 10% normal horse serum in PBS for a further 10 minutes. Slides were stained using sheep anti FAP (R&D, AF3715) for 1 hour at RT. Slides were washed before the application of donkey anti goat HRP (Dako). HRP staining was developed using the ImmPACT DAB Peroxidase (HRP) Substrate (Vector Labs) and counterstained with haematoxylin. Slides were mounted in VectaMount (Vector Labs) before imaging using the Zeiss Axio Scan and analysis using Zen lite 2012 software (both Zeiss, Oberkochen Germany).

### Statistical analysis

Using Prism (Graphpad, La Jolla, CA) software, differences between groups were assessed using the Kruskal-Wallis test with Dunn’s post test for multiple comparisons or the Mann-Whitney test. The Chi-Square test was used to compare categorical characteristics. Correlation of two outcome measurements was assessed with the Spearman rank-order correlation coefficient, using a Benjamini-Hochberg correction for multiple comparisons to exclude type I errors. To assess reliability of outcome variables between multiple staining procedures, intraclass correlation coefficients were calculated using SPSS (IBM, Armonk, NY). A p value <0.05 was considered statistically significant.

## Results

### Patient characteristics

Baseline characteristics of patients from the BEACON (Birmingham) and Synoviomics (Amsterdam) cohorts presenting with symptom duration of three months or less are shown in **Table 1**. In the very early rheumatoid arthritis (VERA) group, 78% already fulfilled the 2010 ACR/EULAR classification criteria for RA at baseline and 22% were first classified as having UA. Eight patients in the VERA group had disease which was ultimately self-limiting. Most patients in the non-RA group had a self-limiting disease, but eight had a persistent disease with a diagnosis other than RA, including four with PsA. As expected, significantly more patients were IgM-RF and/or ACPA positive in the VERA group compared to the non-RA group, and tender and swollen joint counts were significantly higher in the VERA group.

**Table 1:** Baseline characteristics of early arthritis patients.

	VERA (n = 32)	Non-RA (n = 24)	Non-inflammatory controls (n = 7)	P-value
**Age (years)**	51 (43–59)	44 (35–60)	58 (46–69)	0.109
**Female, n (%)**	17 (53.1)	7 (29.2)	3 (42.9)	0.200
**ESR (mm/h)**	32.5 (15.3–61.8)	14 (7–39)	-	0.068
**CRP (mg/l)**	12.3 (4.6–39.5)	9 (6–24)	-	0.403
**TJC68**	8 (2–17)	2 (1–4)	-	0.001
**SJC66**	7 (4–14)	2 (1–3)	-	<0.001
**Symptom duration (wks)**	6 (4–9)	4 (2–6)	-	0.016
**IgM-RF positive, n (%)**	11 (34.4)	0 (0)	-	0.001
**ACPA positive, n (%)**	14 (43.8)	0 (0)	-	< 0.001
**IgM-RF and ACPA both positive, n (%)**	10 (31.3)	0 (0)	-	0.003
**Self-limiting disease, n (%)**	8 (25%)	16 (66.7)	-	0.002
**Diagnoses**	UA-RA 7 (SLD = 6) RA-RA 25 (SLD = 2)	UA 10 (SLD = 7) Gout 5 (SLD = 4) PsA 4 Reactive 3 (SLD = 3) Parvovirus 2 (SLD = 2)		

Values shown are median (interquartile range) or number (percentage). ACPA: anti-citrullinated protein antibodies, CRP: C-reactive protein, ESR: erythrocyte sedimentation rate, IgM-RF: IgM rheumatoid factor, PsA: psoriatic arthritis, RA-RA: patients who fulfil the 2010 ACR/EULAR classification criteria for RA at baseline, SJC66: swollen joint count using 66 joints, SLD: self-limiting disease, TJC68: tender joint count using 68 joints, UA: unclassified arthritis, UA-RA: patients who were classified as UA at baseline, but fulfilling the 2010 ACR/EULAR classification criteria for RA during follow-up, VERA: very early rheumatoid arthritis.

### FAP expression is increased in early arthritis patients who develop RA

**Fig 1:**
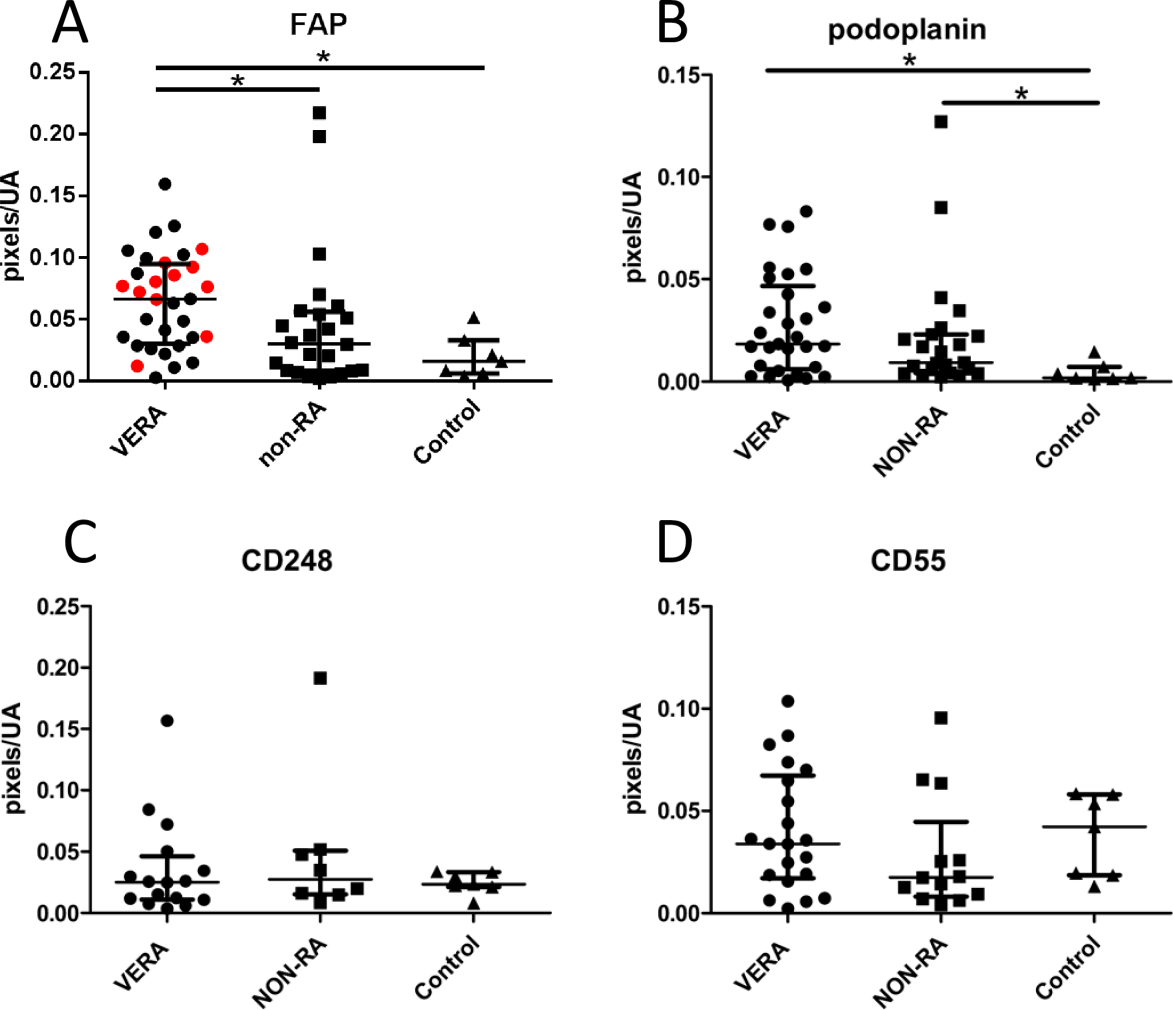
Relationship between stromal markers and diagnostic group in treatment-naive early arthritis tissue. Expression of (A) FAP, (B) podoplanin, (C) CD248, (D) CD55 were measured by pixel counting in synovial regions of interest in uninflamed tissue of patients with mechanical joint symptoms undergoing exploratory arthroscopy (Control), and in baseline samples of early arthritis patients, split into patients fulfilling ACR/EULAR 2010 criteria for RA during follow-up (very early rheumatoid arthritis; VERA) and combined non-RA groups made up of patients with spontaneously resolving arthritis and patients with non-RA persistent arthritis (NON-RA). (A) FAP expression was higher in VERA patients (n = 32) compared to other outcome groups (n = 24) (Kruskal-Wallis p = 0.0036, asterisks denote the results of Dunn’s post-test, *p<0.05). Red data points indicate the subgroup of patients developing seronegative, persistent RA. (B) Podoplanin expression also differed between outcome groups (Kruskal-Wallis p = 0.0062), but there was no significant difference in post-testing between VERA (n = 29) and NON-RA groups (n = 23). (C,D) No significant difference was observed in CD55 (lining; 21 VERA vs 13 non-RA) or CD248 (sublining; 16 VERA vs 8 non-RA) expression. Each dot represents a patient; median bars with interquartile ranges are shown.

In order to test the hypothesis that stromal markers would distinguish diagnostic group, we compared the expression of CD55, CD248, podoplanin and FAP in different clinical outcome groups (**Fig 1**). Baseline expression of FAP was significantly higher in patients fulfilling 2010 criteria for RA regardless of time of fulfilment of criteria compared to the comparison groups (Kruskal-Wallis test p = 0.003, individual comparisons p<0.05 by Dunn’s post test). Significantly higher expression of FAP in the VERA group was seen in the seronegative persistent RA subgroup after excluding RA patients with self-limiting or seropositive disease (p = 0.0035 individual comparisons p<0.05, Fig 1A red data points), suggesting that FAP could be a useful biomarker for seronegative persistent RA. Expression of podoplanin in VERA was increased compared to non-inflammatory control samples (Kruskal-Wallis test p = 0.0062), but no specificity for RA vs other disease outcomes was seen. By comparison greater variability was seen in CD55 and CD248 expression levels, and no difference was seen between different outcome groups.

### Stromal markers do not differentiate between persistent and resolving disease

**Fig 2:**
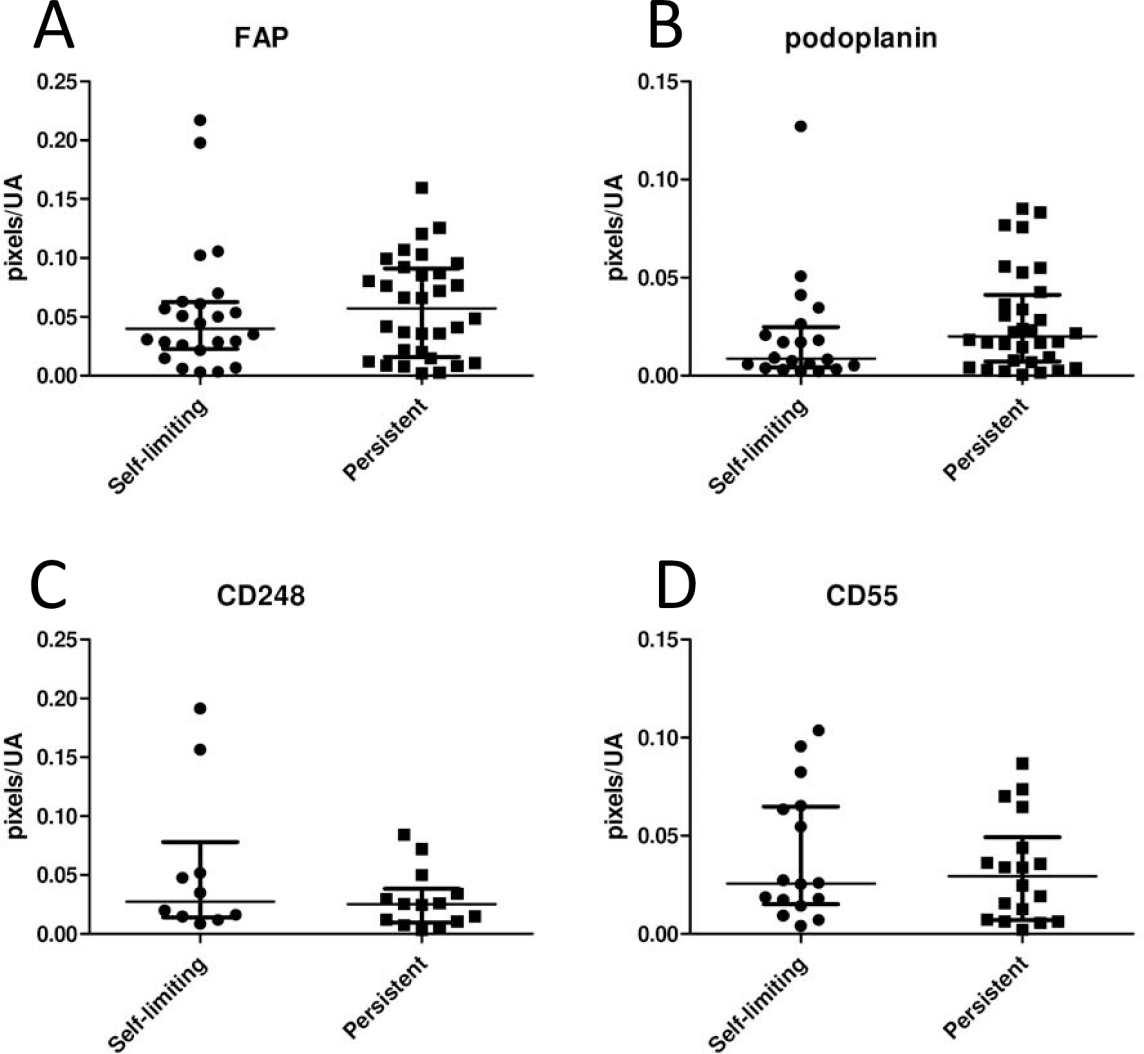
Relationship between stromal markers and prognostic outcome in treatment-naive early arthritis tissue. Expression of (A) FAP (24 self-limiting vs 32 persistent disease), (B) podoplanin (20 self-limiting vs 32 persistent disease), (C) CD248 (10 self-limiting vs 14 persistent disease), (D) CD55 (16 self-limiting vs 18 persistent disease) were measured by pixel counting in synovial regions of interest in baseline samples of early arthritis patients who developed resolving disease (self-limiting) or persistent disease. No significant differences were found. Each dot represents a patient; median bars with interquartile ranges are shown. Significance of the comparisons was determined by the Kruskal-Wallis test.

Having identified an association between FAP expression and the earliest clinically evident stage of RA, we tested the hypothesis that stromal markers would differentiate between the key prognostic outcomes of persistence or resolution in early arthritis. In our combined cohorts, 43% of patients presenting within the first three months of developing symptoms experienced complete resolution of symptoms and signs that persisted in the absence of therapy during follow-up. This proportion is consistent with that seen in previous work in the BEACON cohort [3]. No significant differences were seen between resolving and persistent diseases for any of the stromal markers analysed (**Fig 2**).

### FAP expression is increased in both lining and sublining layers in VERA patients

**Fig 3:**
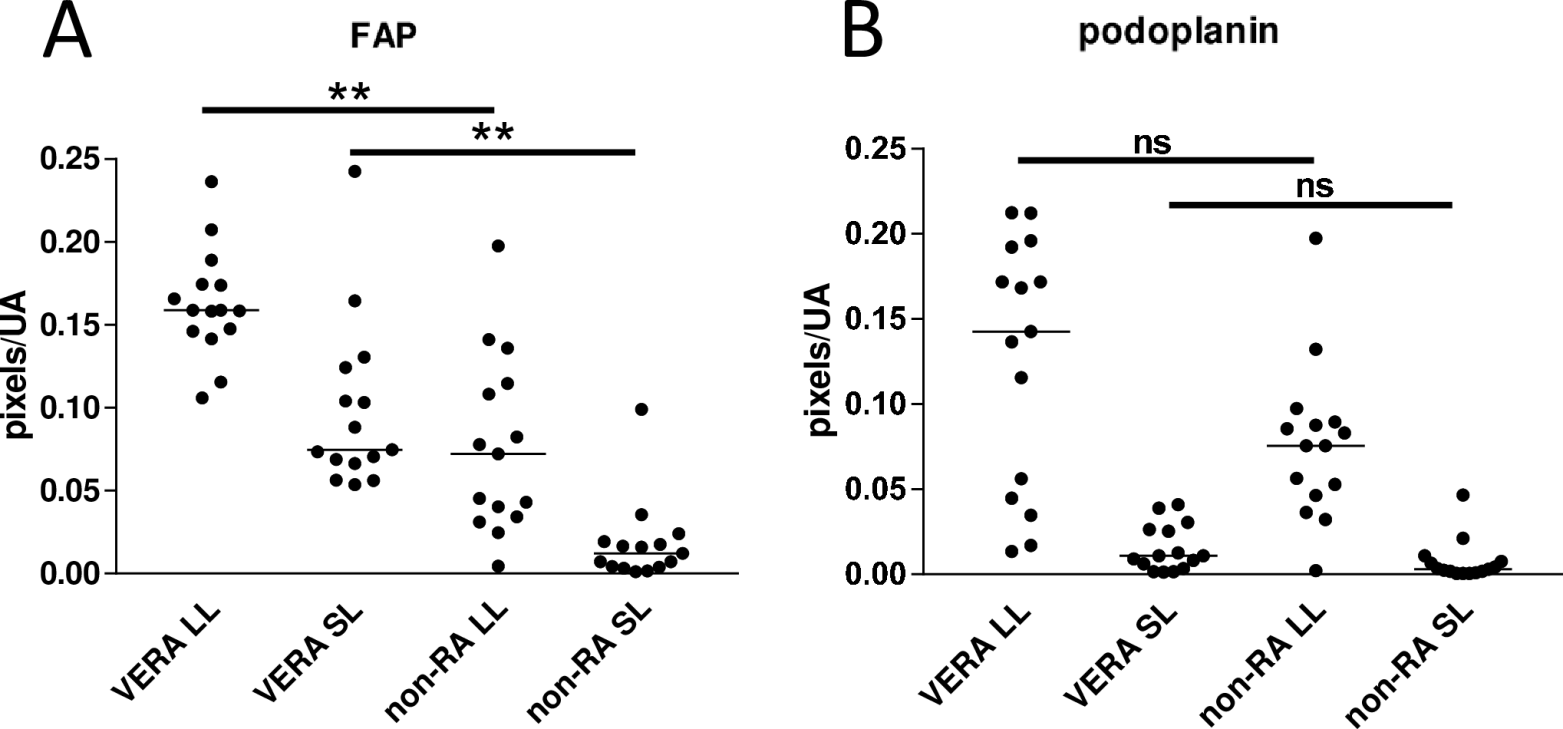
Differences in lining and sublining layer staining patterns of stromal markers with disease. Expression of (A) FAP and (B) podoplanin quantified in lining and sublining layer regions in 15 sections from each group. FAP: Kruskal-Wallis p<0.0001, podoplanin p<0.0001; asterisks denote the results of Dunn’s post-test, **p<0.01).

**Fig 4:**
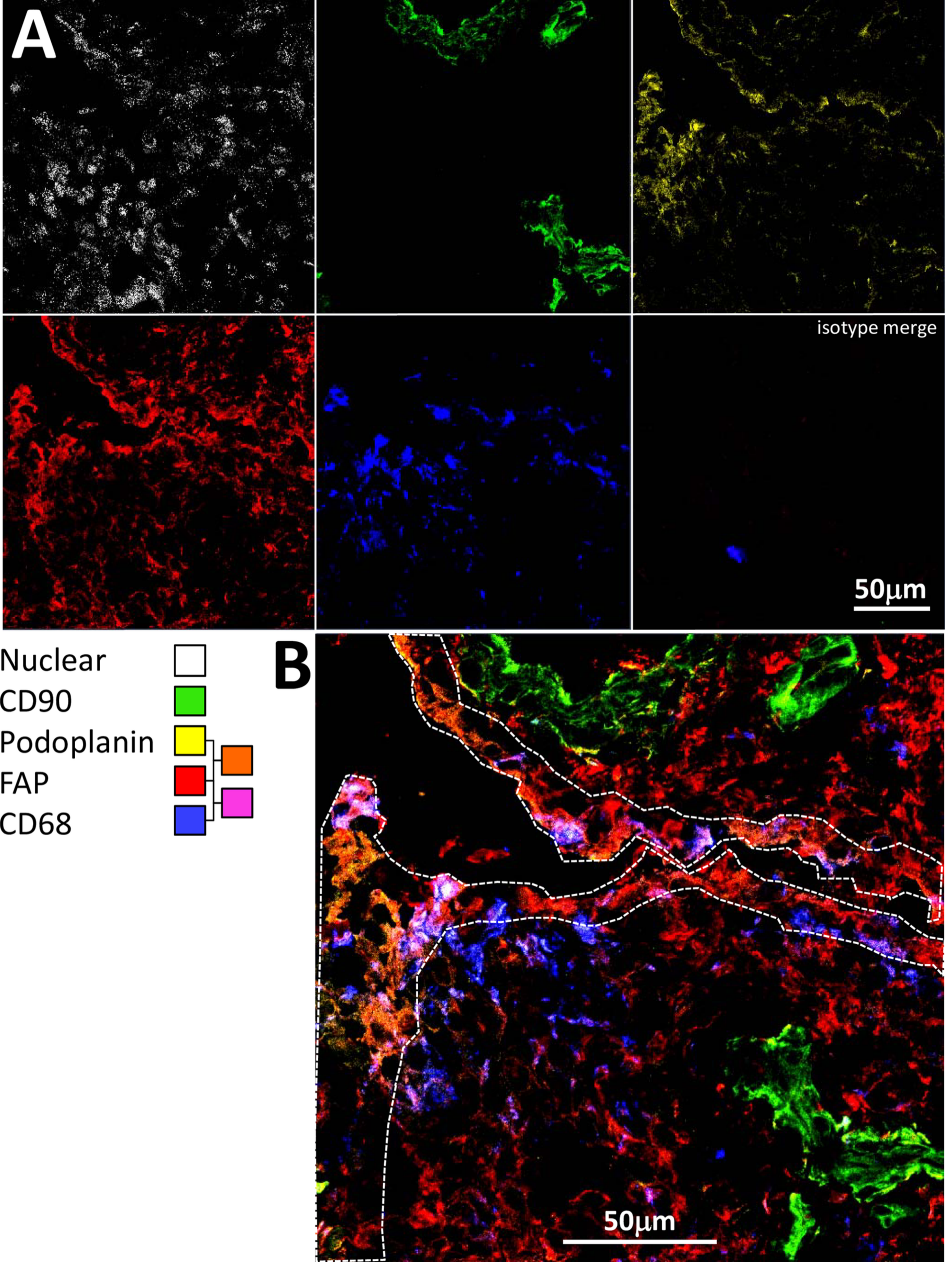
FAP is expressed at high levels throughout the synovium in biopsies of patients developing RA. (A) Multicolour confocal microscopy images are shown for tissue staining at baseline with FAP (F11-24), podoplanin (D2-40), CD68 (Y1-82A), CD90 (Thy-1A1) antibodies followed by secondary agents, and nuclear (Hoechst) stain in a representative patient presenting with RA whose disease persisted. (B) Higher magnification, merged image. The region representing the lining layer is highlighted by a dotted line.

**Fig 5:**
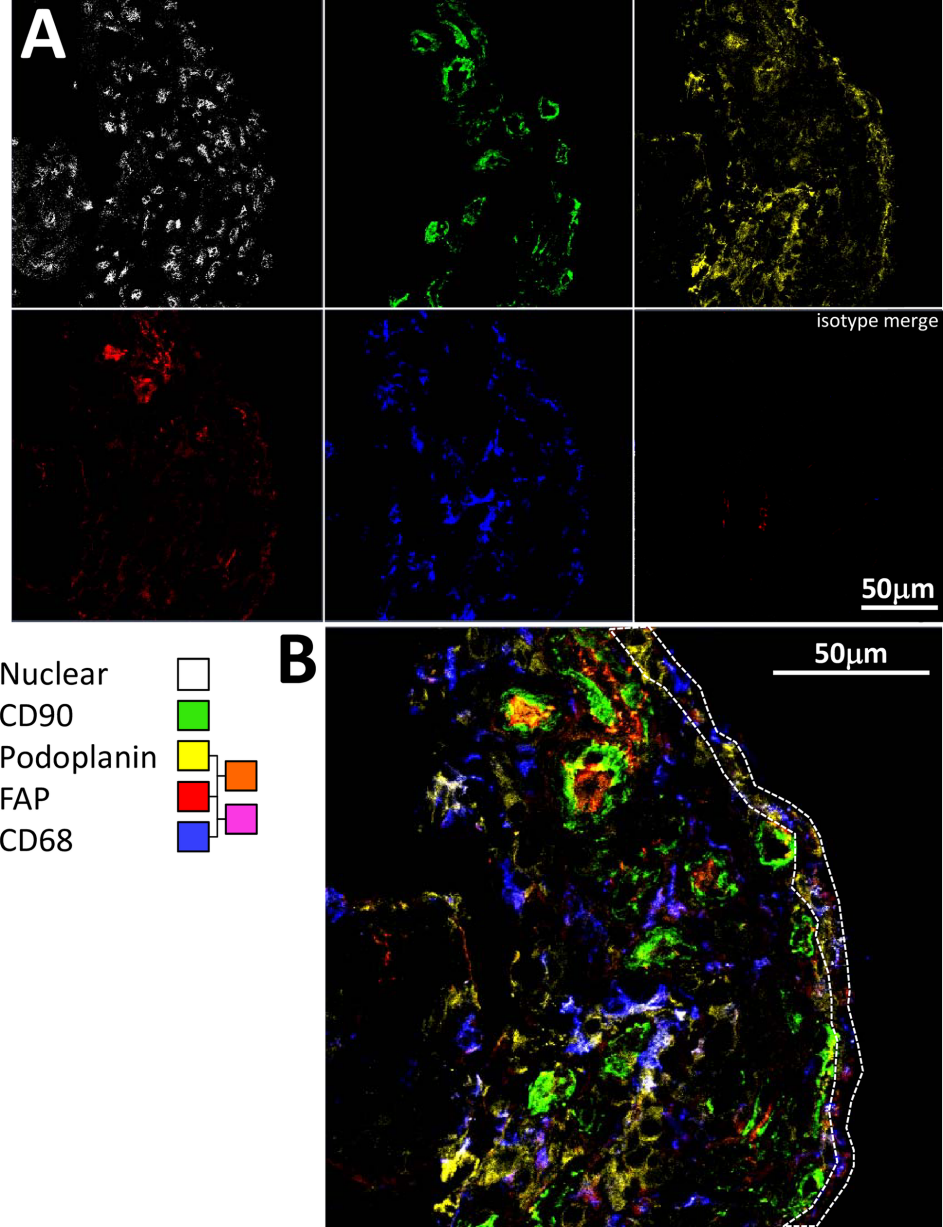
FAP is expressed at low levels in synovial biopsies of patients with self-limiting disease. (A) Multicolour confocal microscopy images are shown for tissue staining at baseline with FAP (F11-24), podoplanin (D2-40), CD68 (Y1-82A), CD90 (Thy-1A1) antibodies followed by secondary agents, and nuclear (Hoechst) stain in a patient with unclassified arthritis whose disease spontaneously resolved. (B) Higher magnification, merged image. The region representing the lining layer is highlighted by a dotted line.

**Fig 6:**
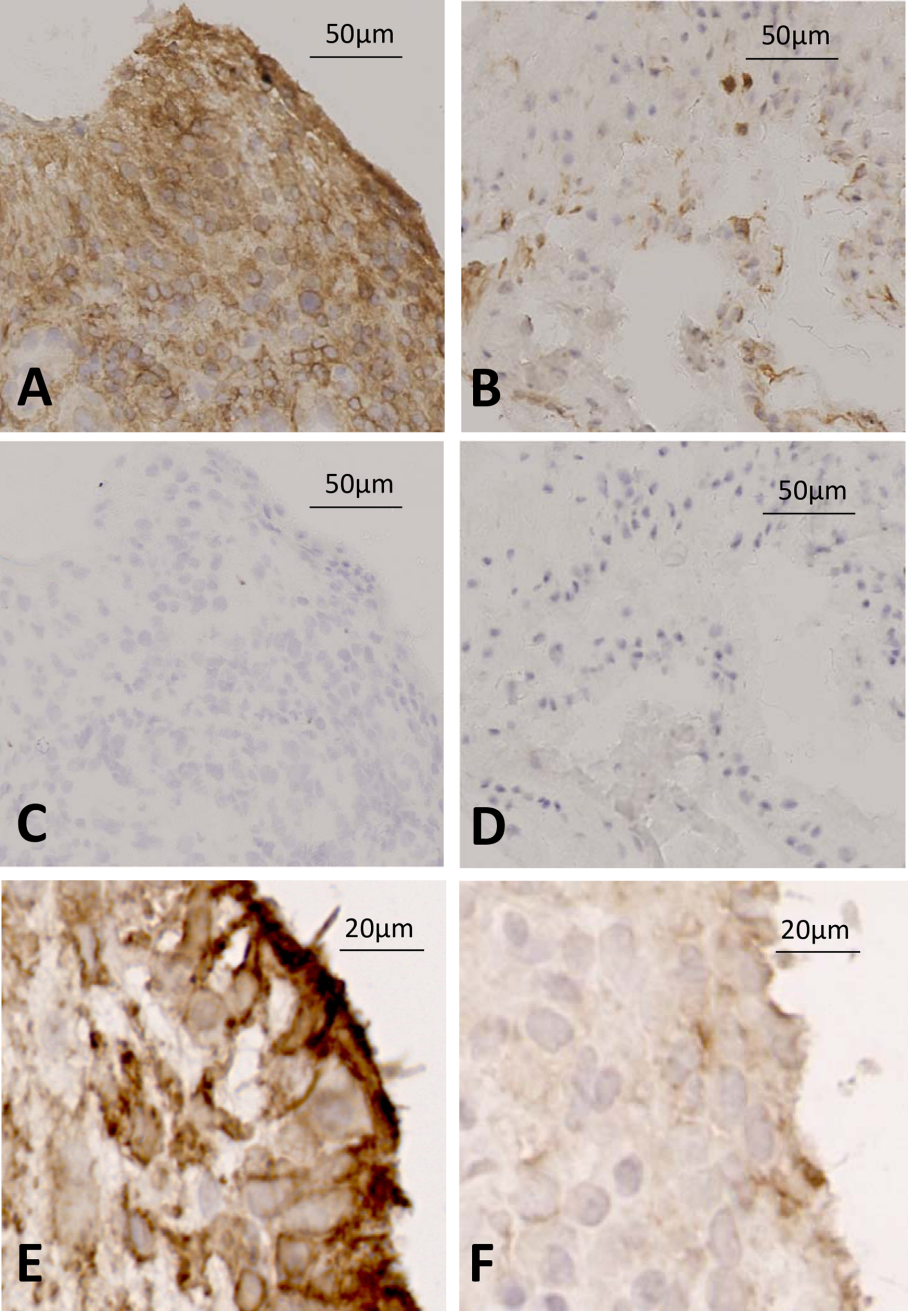
High expression of FAP in tissue from patient developing RA compared to UA control. Immunohistochemistry was used to stain for FAP positive cells using a sheep anti-FAP antibody in representative tissues from patients developing RA and non-RA disease. (A low power, E high power) FAP staining in patient developing RA vs (C) isotype control; (B low power, F high power) FAP staining in patient with undifferentiated arthritis vs (D) isotype control.

In early RA, strong FAP and gp38 staining was seen in the lining layer (Fig 3), but a network of FAP positive cellular staining was also seen in the sublining layer that was not seen in non-RA disease. Quantification of lining and sublining layer staining (**Fig 3**) demonstrated significantly greater sublining FAP staining in early RA, confirming qualitative observations using multicolour immunofluorescence staining (**Fig 4 and Fig 5**). Fluorescence staining for FAP was validated using immunohistochemistry in sections from cohort patients; representative images are shown in **Fig 6**.

### Tissue FAP expression does not correlate with clinical or ultrasound variables

Because FAP expression levels appeared to be associated with diagnostic outcome, we tested whether FAP levels correlated with clinical variables including swollen and tender joint counts, erythrocyte sedimentation rate (ESR), C-reactive protein (CRP) and symptom duration. In the entire cohort of patients with disease duration of three months or less (n = 56), FAP expression levels demonstrated no correlation with these clinical variables after Benjamini-Hochberg correction for multiple comparisons. Within the 23 individuals from Birmingham who had undergone ultrasound scanning of the biopsied joint, there was no correlation between FAP staining and either greyscale or power Doppler semiquantitative ultrasound grading scores.

## Discussion

The most prominent stromal cell of the ST is the fibroblast-like synoviocyte (FLS) [7, 32]. We previously showed that CD55 is a defining marker for FLS in the intimal lining layer where it can mediate contacts with CD97 on other immune cells and may be of primary importance in maintaining and amplifying synovial inflammation [13, 14, 33, 34]. Other molecules markedly expressed by FLS in ST of patients with established RA are CD248 (also known as endosialin or TEM1) and podoplanin (gp38) [16, 21]. CD248 is expressed by FLS in synovium from patients with established PsA and RA, but is only weakly present in synovium from individuals with non-inflammatory knee problems [16], and is believed to play a role in tissue remodelling by increasing proliferation and migration [35]. FAP, a cell surface-bound, type II transmembrane glycoprotein belonging to the family of serine prolyl oligopeptidases, is expressed by activated fibroblasts associated with the granulation tissue of healing wounds and stroma of epithelial cancers. It has also been shown that FAP is strongly expressed in rheumatoid synovium [17]. Of these markers, podoplanin and FAP were found to be upregulated in early RA ST compared to tissue of healthy controls, but only FAP expression appeared to differentiate from other forms of inflammatory joint disease.

We found no correlation between FAP levels and clinical indices of disease activity. This may in part be a consequence of exploratory analysis in a pilot study with moderate numbers. However, it may not be reasonable to expect events in a single joint to reflect pathological events at a systemic level. Existing data show that stromal cell activation profiles are specific to organ and joint location[36, 37]; furthermore the role of stromal cell activation in such early disease may relate to tissue remodelling to accommodate cellular infiltrates and the assembly of pannus tissue capable of mediating joint damage [10, 22]. These pathological processes may not necessarily be reflected in markers of systemic inflammation, as evidenced by longstanding observations of progression of erosion despite apparent clinical remission [38]. Imaging studies have shown that persistent joint inflammation may account for progression of joint damage [39, 40]. We therefore investigated the link between synovial hypertrophy, Power Doppler enhancement and stromal marker expression. There was no correlation between local ultrasound indices and stromal markers, however we have to regard these data with caution given the limited number of patients with such data available. Larger ongoing studies are addressing this hypothesis.

FAP is strongly expressed in ST of patients with destructive RA [17]. It is a surface glycoprotein with both ectoenzyme and transmembrane signalling properties. Ospelt et al. blocked the serine protease ectoenzyme function of FAP in fibroblasts and in the SCID model of fibroblast co-implantation, resulting in increased matrix metalloproteinase production and cartilage breakdown [19], suggesting a protective role of FAP. However subsequent studies have uncovered multiple additional roles of FAP. Wäldele et al. knocked out FAP in the human tumour necrosis factor (TNF) transgenic model of arthritis, resulting in decreased matrix destruction with no effect on synovial hyperplasia or bone erosion; decreased adhesion to cartilage suggested that surface interactions with β1 integrins on the cell surface was a likely mechanism [41]. Various transmembrane signalling pathways have been implicated in the non-proteolytic activity of FAP; in bone marrow stromal cells FAP regulates cellular migration via modulation of RhoA, whilst in epithelial cells regulation of PI3Kinase and Ras/ERK signalling have been demonstrated [42, 43]. In our study cohort, we showed a strong expression of FAP in ST of RA patients early in their disease course. High expression of this marker in both lining and sublining regions of the ST indicates the involvement of dysregulated extracellular matrix remodelling in the early stage of arthritis that could represent a potential therapeutic target in early disease. Nuclear medicine imaging of FAP is already under development, facilitated by the development of monoclonal antibodies in oncology [44]. Furthermore, the recent observation that FAP positive cancer associated fibroblasts are present in the peripheral blood of metastatic breast cancer patients may throw interesting light upon the ability of synovial fibroblasts to spread to distant sites in mouse models of arthritis [45, 46]. Placing our findings in this context, FAP and other stromal markers may have significant roles to play as potential therapeutic targets.

RA FLS show a gene expression profile reminiscent of myofibroblasts, and cells of the intimal lining layer in RA have been found to express α-smooth muscle actin (α-SMA) and type IV collagen [47, 48]. It has therefore been suggested that RA FLS can undergo a process resembling epithelial-mesenchymal transition (EMT), whereby static epithelial cells lose cell-cell contacts, acquire mesenchymal features and manifest a migratory phenotype. This phenomenon is common to early developmental processes, tissue repair, fibrosis and carcinogenesis. Both podoplanin and FAP are known to be involved in EMT and are reported to be highly expressed in cells of the intimal lining layer in RA, with little expression in osteoarthritis synovium [21, 49]. The role of podoplanin in inflamed synovial tissue is unclear, but it is possible that it could relate to interactions with infiltrating leukocyte sub-populations, as seen in podoplanin expressing fibroblast-like reticular cells and lymphatic endothelium of the lymph node [20]. Ultimately this could favour the formation of ectopic lymphoid structures [50]. Since we found upregulation of podoplanin in ST of patients with early RA, the involvement of this marker in an EMT-like differentiation of RA-FLS into myofibroblasts could be of importance in the earlier stages of arthritis.

One of the challenges in the treatment of early arthritis patients is initiating patient tailored treatment as early as possible [51–53]. Personalized medicine in this patient group is aimed at remission, thereby preventing joint destruction and optimizing functional outcome with a minimum of potential harmful side-effects. Despite the importance of making an early diagnosis, in over 40% of rheumatology patients no diagnosis can be made at presentation [3], indicating a need for new diagnostic and prognostic markers. Our findings suggest that synovial stromal marker analysis could play a role alongside other tissue markers in the guidance of treatment decisions in early arthritis patients where outcome is not possible to predict using existing clinical variables [6]. Given these preliminary results, both validation in larger cohorts and combination with other variables are necessary in order to provide clear guidance for clinicians. Our findings and those of others mandate more extensive studies of candidate tissue markers alone and in combination for the prediction of diagnosis and prognosis in larger cohorts of patients with early disease [54]. It would also be useful to examine novel tissue markers in the synovium of individuals at risk of developing RA in order to build upon existing studies using conventional histology, leukocyte and adhesion markers [55].
